# Influence of wind energy utilization potential in urban suburbs: a case study of Hohhot

**DOI:** 10.1038/s41598-021-90499-7

**Published:** 2021-06-02

**Authors:** Wang Wenxin, Chen Kexin, Bai Yang, Xu Yun, Wang Jianwen

**Affiliations:** 1 College of Energy and Power, Inner Mongolia University of Technology, Hohhot, China; 2 College of Civil Engineering, Inner Mongolia University of Technology, Hohhot, China; 3 Inner Mongolia Laboratory of Utilization Mechanism and Optimization of Wind Energy and Solar Energy, Hohhot, China

**Keywords:** Civil engineering, Energy science and technology, Engineering

## Abstract

Given the increasing trend of using wind energy in cities, the utilization of distributed wind energy in cities has been widely concerned by researchers. The related research on the micro-site selection of wind turbines, a sub-project of the Task27 project of the International energy agency, was continued in this paper. The wind speed data of an observation station near Hohhot, Inner Mongolia, with a range of 10–19 m were collected. The evaluation included wind direction, Weibull parameter characteristics, and turbulence intensity. The potential energy output in 10 different heights was estimated using commercial horizontal and vertical axis wind turbines of the same power. Results showed that the following: the three-parameter Weibull distribution model can well describe the statistical properties of the wind speed in this site. The wind speed distribution model constructed from extrapolation parameters reflects the wind speed statistical properties out of detection positions to a certain extent. The wind energy density of the vertical axis wind turbine is slightly lower than that of the horizontal axis wind turbine. Furthermore, more power can be generated from March to May.

## Introduction

The development and utilization of wind energy are an important measure to alleviate the energy shortage and adjust the energy structure. The latest data released by International Energy Agency indicate that the annual growth rate of global offshore wind power is nearly 30% from 2010 to 2018, and that of onshore wind power is nearly 10%^1^^.^ In 2018, China’s offshore wind power installed capacity increased by 1.6 GW, while the onshore wind power installed capacity expanded from 14 GW in 2017 to 19 GW; it now enters the forefront of the market^2^. However, the current onshore wind power generation cannot fully reach the level expected by the sustainable development plan. By 2030, an annual increase of 12% of the power generation should be guaranteed, and more powerful measures are needed to actively develop wind energy. Many researchers choose the indicators in the process of the microscopic location as the focus to research the change rules of urban wind environment, the distribution models, through numerical simulation, field test and statistical methods. The causes of strong turbulence and wind resource assessment optimization, to summarize research framework, wind resource assessment method, and to achieve practical experience data of multiple location by numerical simulation, field tests and statistical inductive method^3–9^.

An accurate description of wind speed and direction is the premise for the accuracy of micro-site selection in urban environments. The use of probability statistics is generally accepted as a method for characterizing wind speed and direction. Some probability and statistical models and their variants are widely used in the study of wind speed statistical characteristics. Since then, new methods of model modification, parameter estimation, and goodness test have been proposed and have shown good applicability in describing local wind resources^10–19^. In 1951, Weibull^20^ explained the powder distribution in detail and established three-parameter Weibull distribution model, including the location parameter, on the basis of double-parameter Weibull distribution. The model was used to analyze a large amount of data. The results showed that the three-parameter Weibull distribution model had good applicability for small sample test. In the wind load calculation of buildings, the Weibull distribution function was first applied to the statistical expression of wind^21^. The Weibull distribution function was then introduced to wind resource assessment. Researchers worldwide have studied the relevance and accuracy of the Weibull distribution to determine the probabilistic statistical characteristics of wind speed^22–32^. Some scholars changed the Weibull function model to match the actual wind speed, such as the upper-truncated Weibull distribution^33^ and inverse Weibull distribution^34^. For the Weibull distribution model alone, the two- and three-parameter Weibull models have different performances in describing the wind environment, and the applicability of the two has been the focus of research and discussion. Stewart and Essenwanger^35^ compared the applicability of the two- and three-parameter Weibull models in describing wind speed frequency based on data from more than 40 stations near the ground. They concluded that the three-parameter Weibull models are more flexible and appropriate than the two-parameter model. Piotr Wais^36^ believed that the three-parameter Weibull distribution is seldom applied in the wind power industry, which may lead to the underestimation of the distribution model’s role in wind resource estimation to some extent. Given the lack of comparison with other probability models and detailed analysis in space and time, the role of the three parameters in wind energy evaluation may be ignored to a certain extent. Van Der Auwera^37^ estimated the wind power density in accordance with the three-parameter Weibull distribution and analyzed parameters at different heights. Emeis^38^ studied the wind speed data of 20–140 m measured by Sodar at different landforms and mountain peaks in Germany and researched the parameter variations at different heights. In 1988, through theoretical derivation, Huwenzhong^39^ estimated the approximate formula of parameters and its precision in the Weibull distribution function from the mean wind speed and its standard deviation. In 1996, Wang^40^ proposed a hybrid frequency model that was consistent with wind speeds in inland areas, and its accuracy was better than that of the two-parameter Weibull model. However, the maturity of the Weibull wind frequency probability model is mainly reflected in the applicable evaluation of large wind turbines in large areas on land and at sea. Actual data and methods to discuss the evaluation of wind energy in urban areas are lacking.

In the Task27 project conducted by the international energy agency, researchers from various countries paid close attention to the micro-site selection of wind turbines in complex urban built environments. In Inner Mongolia University of Technology, Wang et al. carried out a series of work on urban wind energy utilization topics, such as urban boundary layer and microscopic site selection, rooftop wind turbine utilization, turbulence model of building wind environment, and the effect of obstacles on turbulence. The three following aspects were considered. First, the accuracy of different turbulence models in building wind environment numerical simulation was compared^41^. The numerical analysis method of wind turbine micro-site selection in urban buildings was summarized^42^, discussed, and analyzed^43^. The specific installation height of wind turbine at different types of roofs^44^ was also discussed and analyzed. Second, a new method for numerical research and analysis of micro-site selection of rooftop wind turbine based on the urban atmospheric boundary layer theory was proposed^45^. The power of special rooftop wind turbine for buildings in the city was predicted. Third, the influence of non-architectural factors in the urban turbulent environment, such as the disturbance effect of different inflow angles, wind profile, surface roughness, and hedge wall, on the micro-site selection of the wind turbine was further studied. The effect of flow field characteristics was also simulated^46^.

Site selection analysis is carried out for specific urban environmental areas to enrich the measured data of urban wind energy. Based on the work of the research team, this study continues the research topic of Task27 on wind energy potential and urban wind energy. The mechanism of wind energy use in Inner Mongolia Autonomous Region is explored. The key laboratory of renewable energy base, which was built by the ZephIR sonde and laser radar wind mast perennial ground meteorological observation system, is optimized. ZephIR laser radar is the main test method and data source used. It allows 11 Locations near the ground to be vertically observed the features of the wind speed and turbulence characteristics. The Weibull probability model is used to fit the cumulative probability distribution function of each height. Least square method is used to estimate the shape, scale, and position parameters. Finally, the wind energy density of the entire year and each month is calculated, and the turbulent environment around the observation site is analyzed. According to the parameters of wind turbine micro-site selection, some suggestions are introduced for the selection of urban near-surface and micro-wind turbine to provide a favorable reference for related projects and research on the utilization of wind energy in the future.

## Methodology

### Urban wind profile

The wind profile formula, Eq. (1) of exponential law, was proposed by Hellman in the 1820s. After continuous improvement^47^, it could further describe the atmospheric boundary layer within the range of 30–300 m^48^:

1 $$ V_{h} = V_{g} \left( {\frac{h}{{h_{g} }}} \right)^{a} $$

where *V*_*h*_ is the average velocity at the height *h*_*g*_, m/s; *V*_*g*_ is the average velocity at the reference height *h*_*g*_, m/s; *a* is the wind speed profile index or wind shear index, which is determined by the surface roughness height.

For the urban terrain with relatively dense and low-rise buildings, some Japanese scholars further proposed that the urban rough sub-layer conforms to the logarithmic wind profile^49^. Its expression is presented in Eq. (2):

2 $$ v(h) = \frac{{v^{*} }}{k}\ln \left(\frac{{h - h_{0} }}{{h_{0} }} \right) $$

where: v* is the friction velocity, m/s; *h*_*0*_ is the aerodynamic roughness height of the ground, m; *h* is the vertical height of the research point from the ground, m; *k* is the Carmen constant, generally 0.4.

### Wind speed distribution

In general, the Weibull distribution function applied to statistical analysis of wind speed mainly includes two and three-parameter models. The two-parameter model is shown in Eq. (3):

3 $$ f(v) = \frac{k}{c} \left(\frac{v}{c}\right)^{k - 1} e^{ - (v/c)k} $$

where *v* is the wind speed, *v* > , 0, *k* > , 0; and *c* > 0. The dimensionless factor *k* determines the shape of the curve and is called the shape factor. Parameter *c* is the scale parameter. The three-parameter Weibull distribution function adds the position parameter *v*, which considers the value of zero wind speed and determines the starting position of the distribution curve. Equation (4) presents the distribution function model:

4 $$ f(v) = \left(\frac{k}{c}\right)\left(\frac{v - u}{c}\right)^{k - 1} e^{{ - \left(\frac{v - u}{c}\right)^{k} }} $$

Parameter estimation methods include moment estimation and maximum likelihood estimation. Considering the precision of parameter estimation and the complexity of calculation, this study uses the nonlinear regression equation to construct the probability function model of three-parameter Weibull distribution. The values of parameters *u*, *k*, and *c* are estimated by the least square method. The parameter estimation process is described as follows.

First, the probability distribution function in Eq. (4) is transformed into the cumulative probability density function Eq. (5):

5 $$ F(v) = 1 - e^{{\left( - \frac{v - u}{c}\right)^{k} }} $$

Equation (6) is obtained by substituting Eq. (5) into the term and solving the logarithms of both sides of the equation.

6 $$ \ln \left[ - \ln (1 - F(v)) \right] = k\ln (v - u) - k\ln c $$

Ln(v-u) in Eq. (6) is regarded as *X*, ln[-ln(1-F(v))] as *Y*, and the corresponding residual terms and coefficients are regarded as parameter sets. According to the principle of least square, the estimated values of parameters *k*, *c*, and *u* of the original function F(v) are obtained using Eqs. (7), (8), and (9)^50–52^, respectively, as shown below:

7 $$ k = \frac{{\sum {(X_{i} - \overline{X})} (Y_{i} - \overline{Y})}}{{\sum {(X_{i} - \overline{X})^{2} } }} $$

8 $$ c = e^{{\left[\frac{{ - (\overline{Y} - k\overline{X})}}{k}\right]}} $$

9 $$ u = \min \{ \sum\limits_{i = 1}^{n} {[Y_{i} - (\overline{Y} - kX_{i} )} ]^{2} \} $$

### Turbulence intensity (TI)

TI represents the turbulence development intensity and the ratio of the standard deviation of wind speed to the average wind speed. Under the same set of measurements and the specified period, a TI of 10 min is calculated using Eq. (10)^37^:

10 $$ I_{T} = \frac{\sigma }{V} $$

TI is a measure of turbulence that fluctuates with the value of wind speed. It is one of the important parameters that determine the safety level or design standard of wind turbines, and it is also an important part of wind resource assessment in wind farms. The assessment results directly affect the selection of wind turbines.

According to IEC 61400-1 (2019 edition), TI is divided into four grades A+ , A, B, and C (Table 1) to determine the classification of wind turbines.

**Table 1:** Turbulence intensity class.

Turbulence intensity class	Values
A^+^ (designates the category for very high turbulence characteristics)	0.18
A (designates the category for higher turbulence characteristics)	0.16
B (designates the category for medium turbulence characteristics)	0.14
C (designates the category for lower turbulence characteristics)	0.12

### Wind power density

From the subjective perspective of wind energy utilization, the key to determining the wind energy potential of a city is the amount of kinetic energy, which can be converted from the wind speed. See Eq. (11) for the power calculation equation:

11 $$ P_{v} = \frac{1}{2}mv^{2} = \frac{1}{2}\rho Qv^{2} = \frac{1}{2}\rho A_{R} v^{3} $$

According to Eq. (4), the wind energy density based on position parameter *u*, shape parameter *k*, and scale parameter *c* is used to illustrate the wind energy environment conforming to Weibull probability distribution characteristics. The wind energy density equation is shown in Eq. (12):

12 $$ P_{V} = \frac{1}{2}\rho A_{R} \cdot k\frac{1}{{c^{k} }}\int\limits_{0}^{\infty } {v^{3} \cdot (v - u)^{k - 1} e^{{ - (\frac{v - u}{c})^{k} }} dv} $$

where *ρ* is the air density (it's calculated by Eq. (13)) and *A*_*R*_ is the swept area of the wind turbine^53^.

13 $$ \rho = \frac{m}{V} = \frac{12.68}{g} \cdot \frac{273}{{273 + t}} \cdot \frac{p}{1.103} $$

where *g* is the gravitational acceleration, *t* is the temperature, and *p* is the absolute pressure Eq. (14) is obtained by integrating Eq. (12):

14 $$ \begin{aligned} P_{V} & = \frac{1}{2}\rho A_{R} \left ( - \frac{1}{k}\right) \left (e^{{ - \left (\frac{v - u}{c}\right) ^{k} }} kv^{3} + 3cu^{2} \Gamma \left[\frac{1}{k},\left (\frac{v - u}{c}\right) ^{k} \right] \right. \hfill \\  & \quad \left. + 6c^{2} u\Gamma \left[\frac{2}{k},\left (\frac{v - u}{c}\right) ^{k} \right] + 3c^{3} \Gamma \left[\frac{3}{k},\left (\frac{v - u}{c}\right) ^{k} \right]\right)  \hfill \\ \end{aligned} $$

where Γ(v, z) is the incomplete gamma function. The calculation method is described in Eq. (15):

15 $$ \Gamma (v,z) = \int\limits_{z}^{ + \infty } {u^{v - 1} e^{ - u} du} = \Gamma (v) - \gamma (v,z) $$

### Goodness of fit test

The goodness test of parameter estimation and regression analysis in this study mainly focuses on R^2^ and $\chi^{2}$ to test the degree of agreement between fitting value and actual value. The calculation process is shown in Eqs. (16) and (17):

16 $$ R^{2} = 1 - \frac{{\sum\limits_{i = 1}^{n} {(F_{i} - F_{ic} )} }}{{\sigma^{2} }} $$

17 $$ \chi^{2} = \sum\limits_{i = 1}^{n} {\left[\frac{1}{{y_{ic} }}(y_{i} - y_{ic} )^{2} \right]} $$

### Ethics approval and content to participate

The submitted paper has not been published previously, is not under consideration for publication elsewhere, its publication is approved by all authors and tacitly or explicitly by the responsible authorities where the work was carried out.

### Consent for publication

If accepted, it will not be published elsewhere in the same form, in English or in any other language, including electronically without the written consent of the copyright-holder.

## Site description and data analysis

### ZephIR Lidar test system

**Figure 1:**
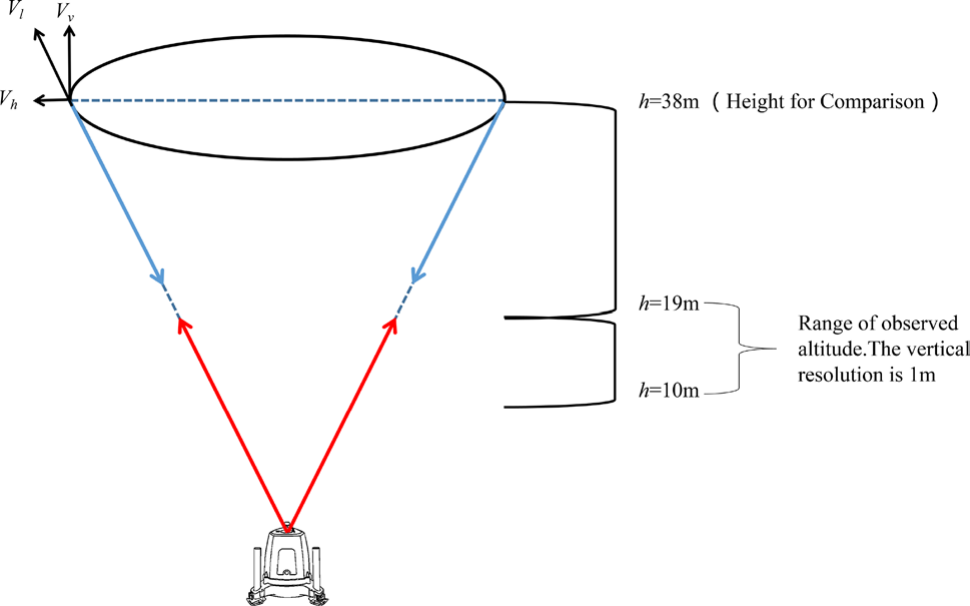
ZephIR Lidar testing principles.

Wind lidar has been widely used in wind resource evaluation. The basic principles of lidar depend on measuring the Doppler shift of radiation from wind-borne natural aerosols, such as droplets, pollen, or dust (the parameters are shown in Table 2). The ZephIR pulse lidar can simultaneously measure at different heights over long distances in space (see Fig. 1 for the testing principle and the setting height of wind speed in this paper). *V*_*l*_ is the radial velocity along the diameter line of strafing cone obtained directly by radar test, whereas *V*_*h*_ and *V*_*z*_ are the velocity components in horizontal and vertical directions, respectively. According to the observation height setting shown in Fig. 1, ZephIR Lidar continuously observes the weather and wind speed for one year and three months, during this period, the observing system will automatically mark the invalid data caused by the signal obscured by floating objects. When the power supply is intermittent or stopped, the data is displayed as a blank group. After removing the above invalid group and blank group data, the integrity of the observation data is about 91.88%.

**Table 2:** Main parameters of ZephIR Lidar.

The parameters of ZephIR Lidar	Parameter values
Measuring range	10–200 m (Lidar survey) 0–10 m (Shipboard weather station)
The length of the probe	± 0.07 m@10 m ± 7.70 m@100 m
Measure the height	The user is configured with 10 heights plus an additional met station for measurement
Sampling frequency	50 Hz (up to 50 measuring points per second)
The average ratio	The actual value is 1 s, with a 10-min average of 0.1 m/s
Wind speed resolution	0.1 m/s
Change the direction of the wind	< 0.5°
Change the direction of the wind	< 1 m/s–80 m/s

### Observation environment

#### Geographical location and building profile observations

**Figure 2:**
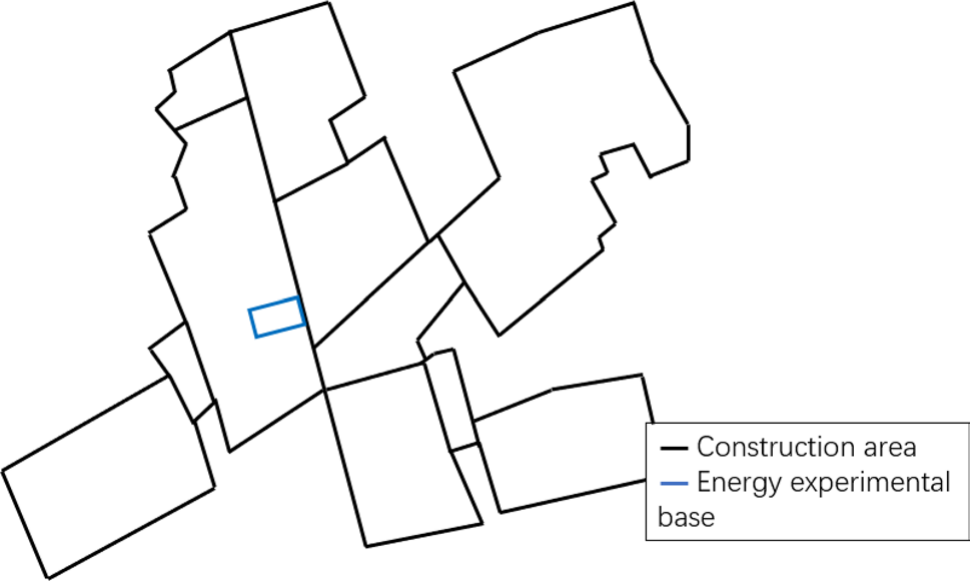
Architectural features of the observation site.

**Figure 3:**
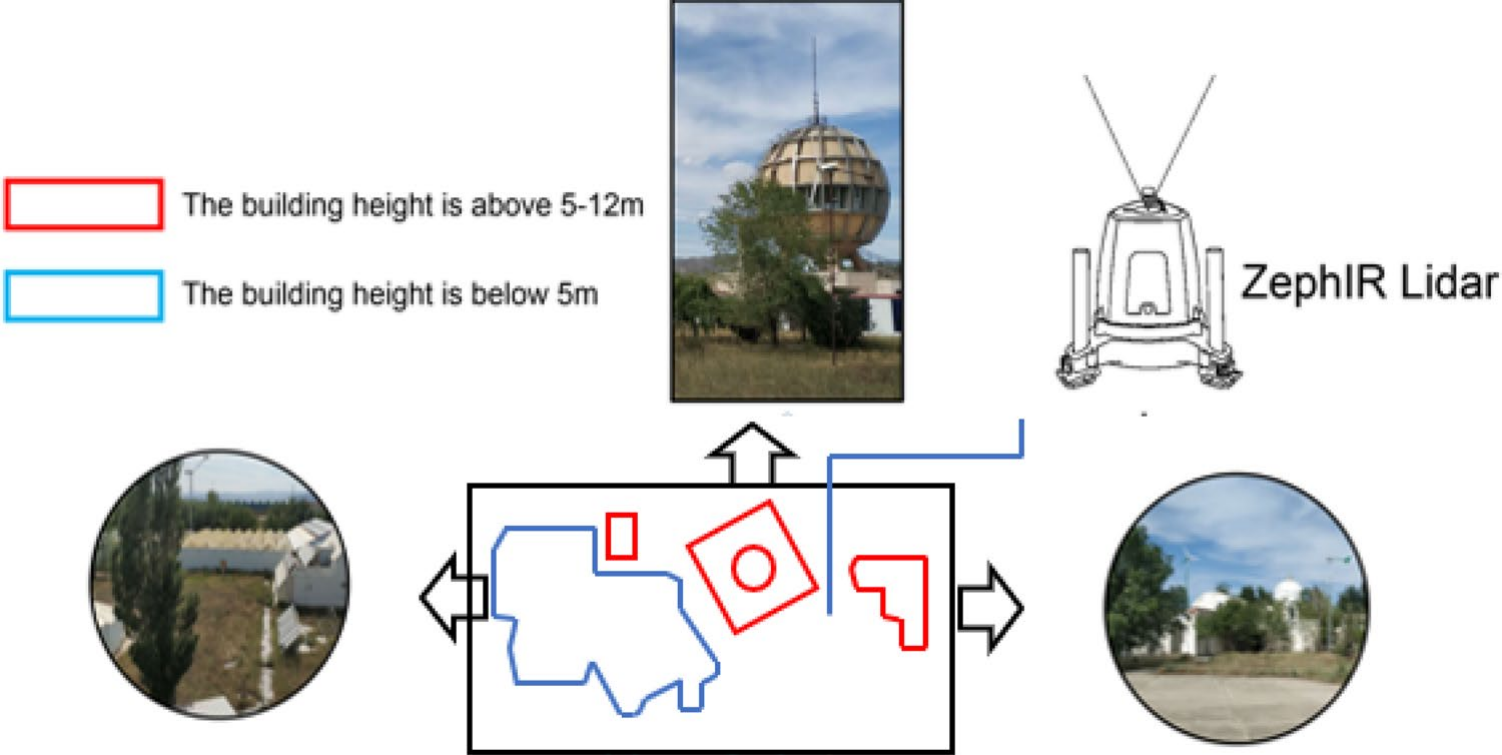
Observation environment was positioned with the ZephIR Lidar.

The lidar observation system is located in the experimental base of the key laboratory of wind and solar energy utilization mechanism and optimization in Inner Mongolia Autonomous Region. The specific locations of the geomorphology, building plane, and observation system are shown in Figs. 2 and 3. The site environment consists of mostly low-rise buildings with a building density of less than 50% surrounded by a single row of aspen poplars and a shrub green belt of approximately 0.75 m. Figure 3 can be seen that the terrain of the observed area in the suburb is relatively open. In this area, the influence of urban heat island effect is more less, the wind speed of is higher than that of the buildings in the urban area, and the wind direction is stable. In addition to low shrubs and lawns, there are no tall obstacles, buildings and trees, which are suitable for the development of wind energy resources. It is a natural wind field in line with the characteristics of most urban suburbs.

#### Meteorological conditions at the observation site

**Figure 4:**
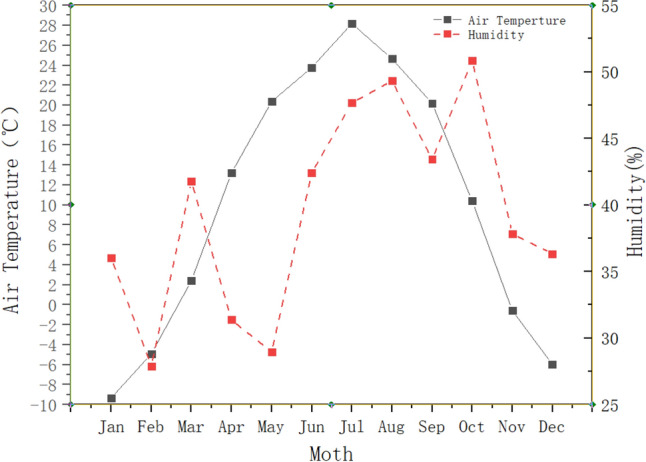
Monthly average changes of temperature and humidity.

**Figure 5:**
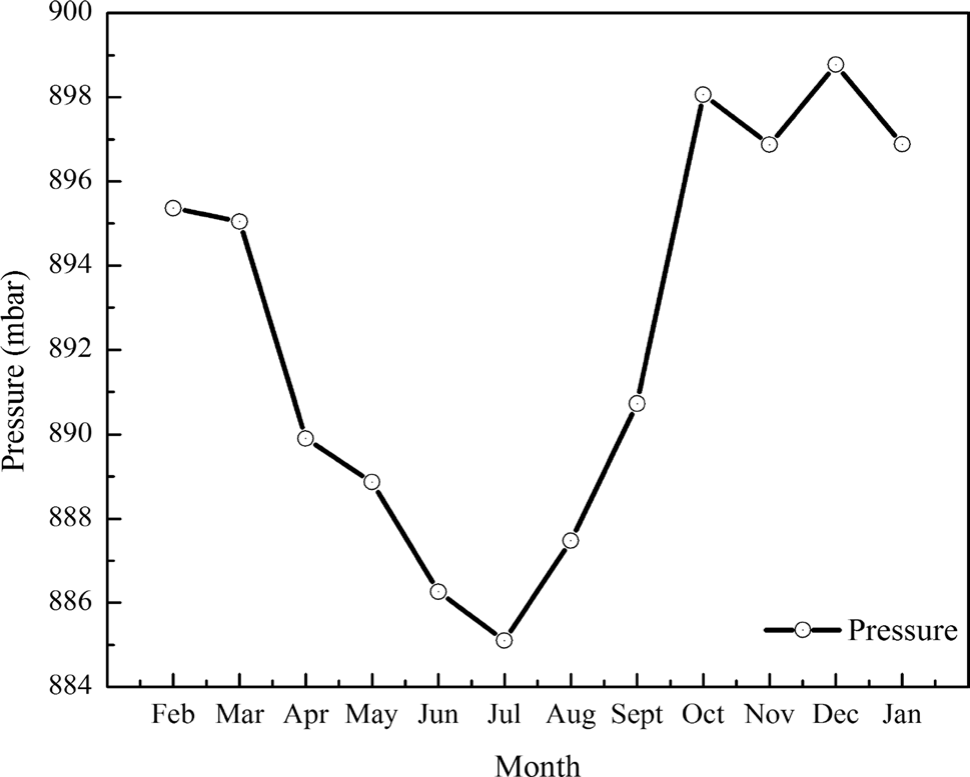
Monthly average variation of atmospheric pressure.

**Figure 6:**
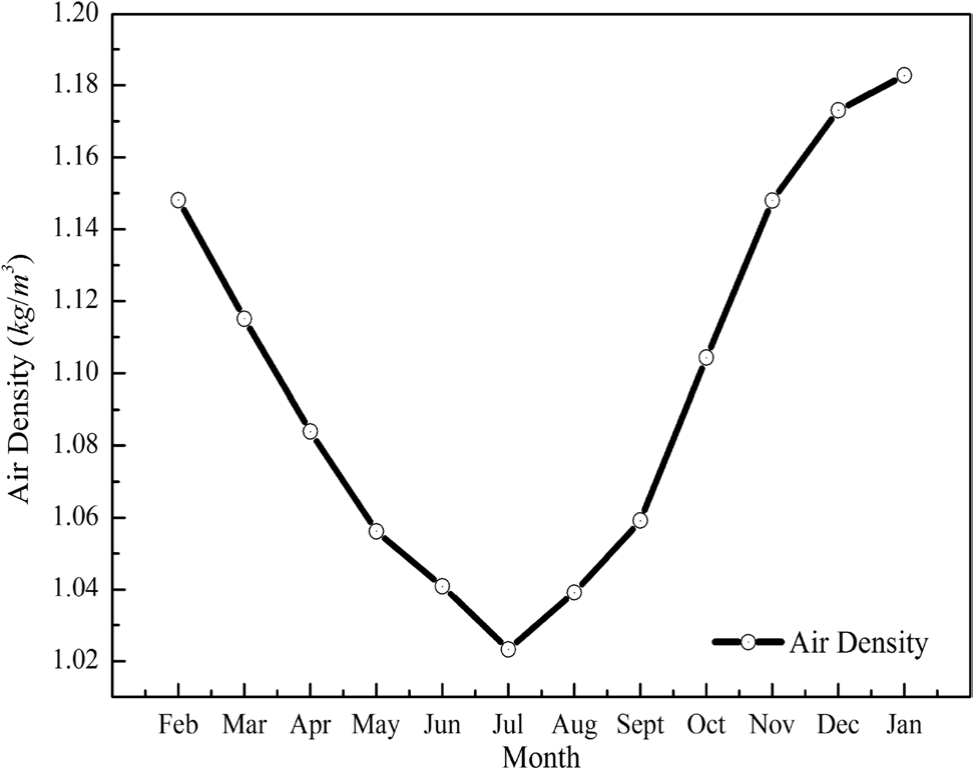
Average change of air density.

Meteorological conditions refer to the hydrothermal conditions of various weather phenomena. The main meteorological conditions of the urban complex environment are wind speed, temperature, humidity, and atmospheric pressure. The parameters related to the utilization of wind energy mainly include wind speed and air density calculated by temperature T Air and atmospheric pressure. The above meteorological parameters are shown in Figs. 4, 5 and 6.

The altitude of the observation site is 1014 m, and the annual average temperature is 10.3 °C. The average atmospheric pressure is 892.4 mbar, and the average humidity is 39.6%. The average air density is 1.09 kg/m^3^, as calculated by Eq. (17). The monthly variation law of atmospheric pressure and air density is basically the same. The air density and air pressure are the lowest in July when the average air temperature is highest. During this period, the air density varies from 1 to 1.2 kg/m^3^, and the air pressure varies from 884 to 900 mbar, both of which are lower than the atmospheric pressure and air density values under standard conditions.

### Characteristics of the wind environment

#### Wind direction characteristics

**Figure 7:**
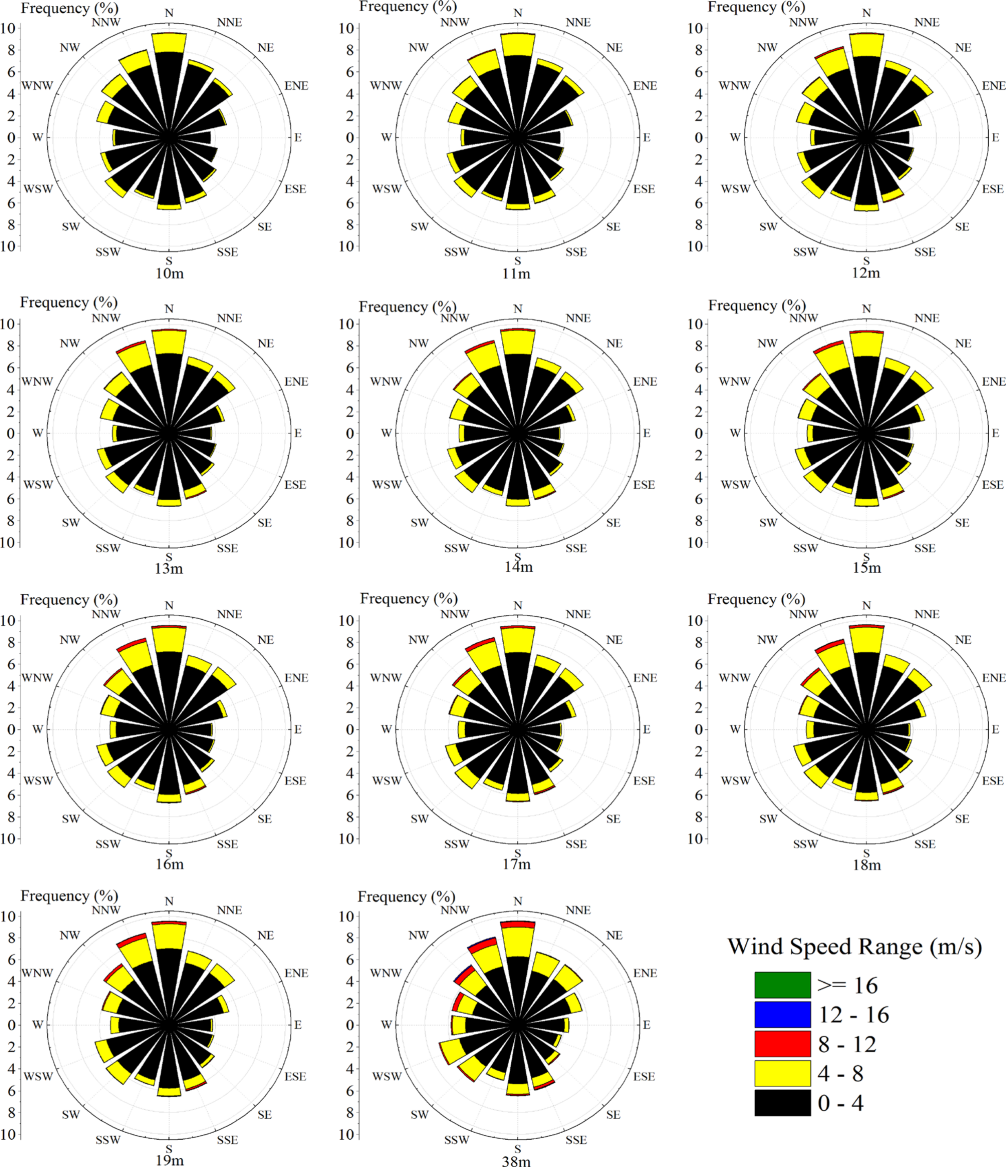
Wind roses at various altitudes.

**Figure 8:**
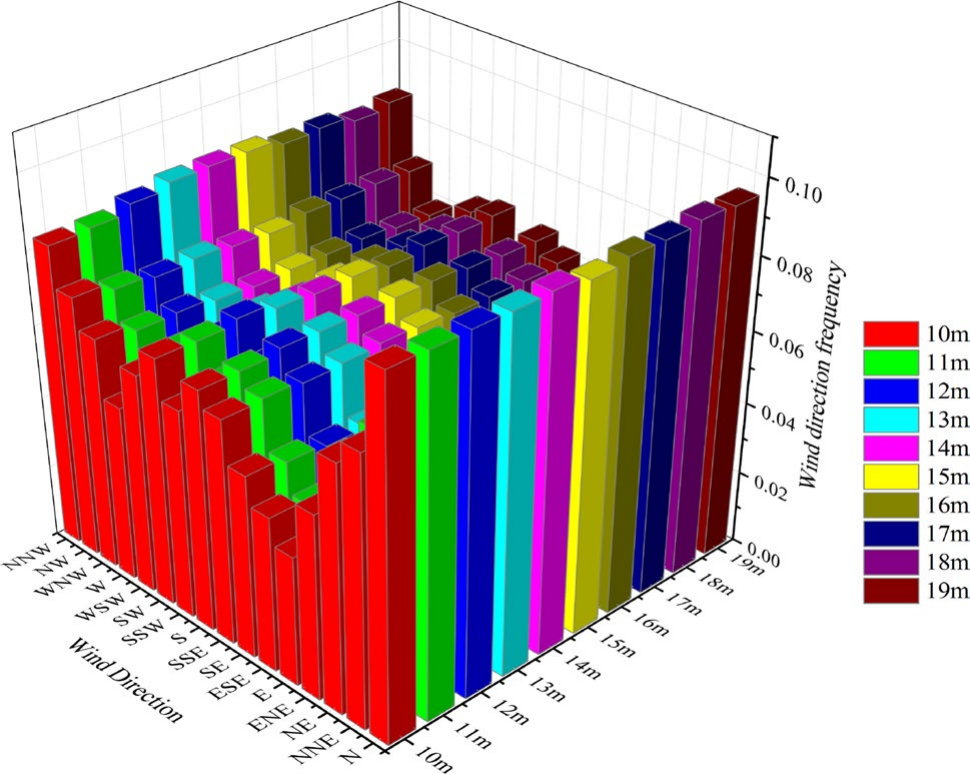
Wind direction frequency variation trend at each observed height.

The change of wind direction is very important for wind turbines, especially vertical axis wind turbines. Figure 7 shows the annual wind direction and wind speed frequency rosettes at heights of 10–19 m. The main wind direction at all observation positions, including the contrast height, is N. However, it is not very different from other minor winds. The direction in which wind occurs most frequently is between two tall buildings. However, in the continuous height range (Fig. 8), no significant difference was found in the wind direction frequency of 10 positions.

#### Wind speed power law characteristics

**Figure 9:**
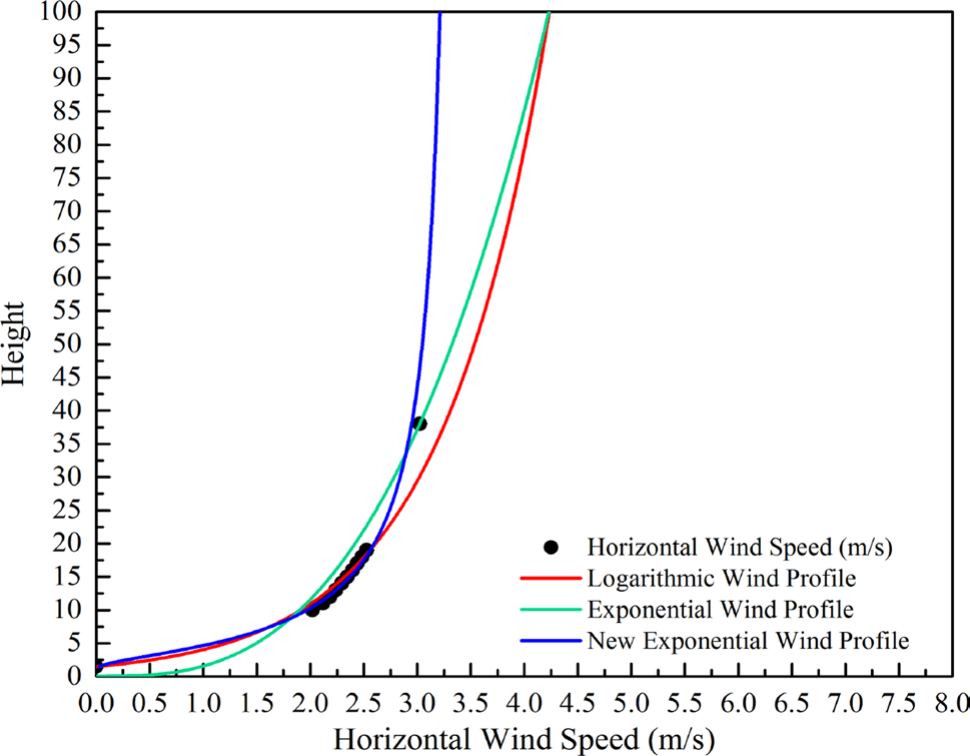
Various forms of wind speed power law changes.

The wind shear coefficients within 10–19 and 19–38 m (Table 2) can be calculated from the Exponential Law (Eqs. 1 and 2). The coefficient value between 12 and 19 m is 0.32, whereas coefficient values of more than 19 and within 10–12 m show significant differences. In comparing the exponential and logarithmic profile curves to simulate wind shear in the vertical direction of wind speed, the exponential law is closer to the actual value than the logarithmic law at 38 m. Furthermore, the exponential law form using the natural constant e as the base is close to the actual average wind speed value (Fig. 9, Eq. (18)). However, the height observed in this paper is concentrated in 10–19 m, so it is impossible to judge the accuracy of its estimation of the horizontal wind speed value above 38 m. However, compared the differences of wind shear coefficient in different height (Table 3), the wind shear coefficient between 12 and 19 m tends to be stable. It can be reasonably assumed that the vertical shear of wind speed may be more hierarchical in the atmospheric boundary layer corresponding to the complex landform in the suburb of the city. However, Eq. (18) shows excellent agreement with the actual wind speed at the observation altitude because of the addition of a correction (x-0.43) to the altitude, and through the coefficients of 3.88 and 5.17, the function is limited and modified at the wind shear section and the atmospheric boundary layer height where the wind shear tends to 0, but its specific role and correlation still need to be combined with the hydrodynamics experiment and theory on the determination of derivation, further discussion will not be made here.

18 $$ V(h) = 3.38 \cdot e^{{\left( { - \frac{5.17}{{x - 0.43}}} \right)}} $$

**Table 3:** Wind shear index within the range of 10–19 and 19–38 m.

Height range	10–11	11–12	12–13	13–14	14–15	15–16	16–17	17–18	18–19	19–38
α	0.49	0.35	0.32	0.32	0.33	0.32	0.31	0.31	0.31	0.26

#### Wind speed statistical characteristic

**Figure 10:**
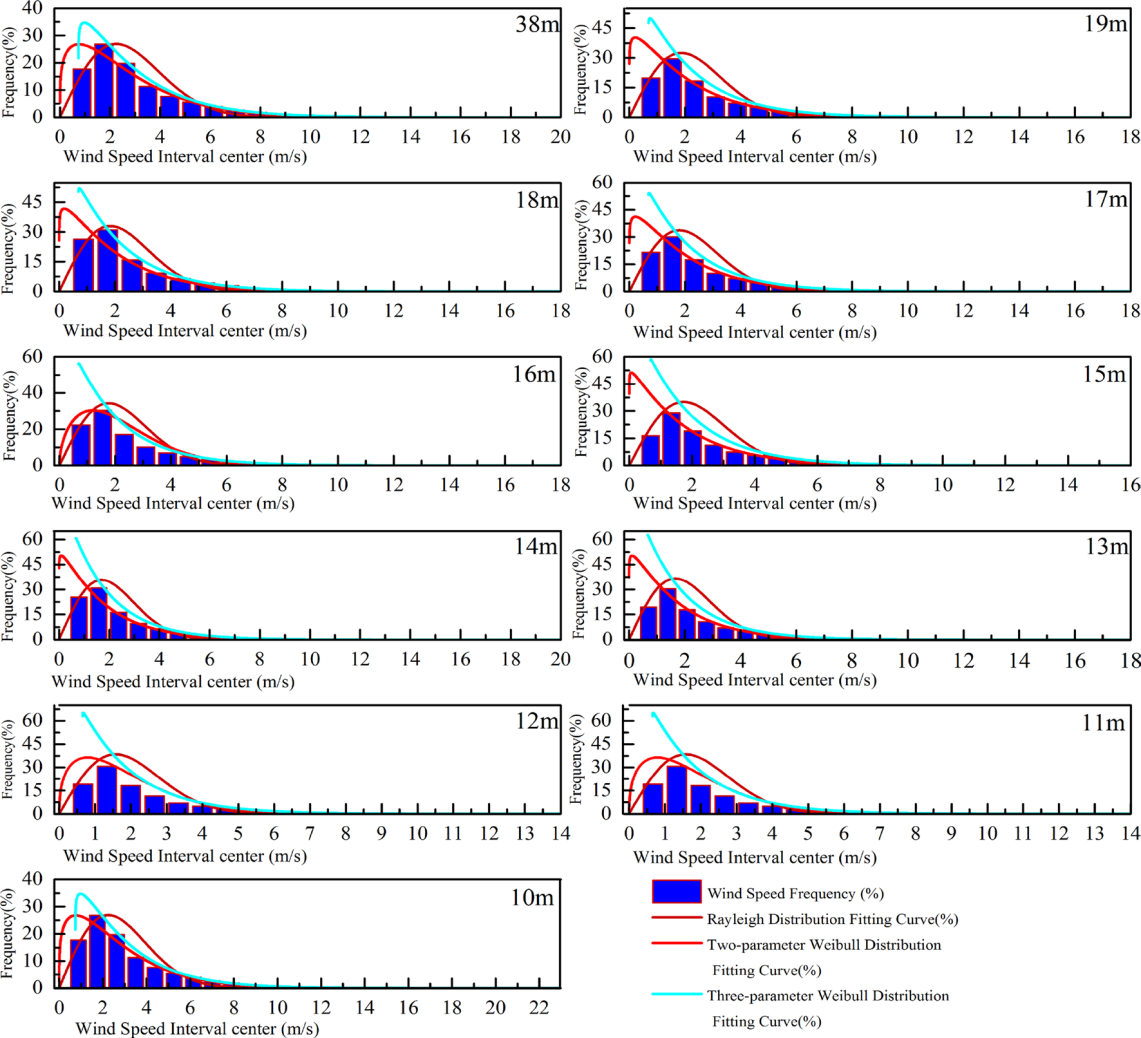
Three type of probability distribution of wind speed at the observed altitude.

In this study, a three-parameter Weibull model is used to analyze the probability distribution of wind speed at different heights (10–19 and 38 m) during the observation period. The probability statistics and nonlinear regression analysis are presented in Fig. 10. The parameters obtained from the least square method (Eqs. (7), (8) and (9)) are shown in Table 4. Therefore, this paper compares the differences between the Rayleigh distribution, the two parameter Weibull distribution and the three parameter Weibull distribution function model with the actual probability distribution, as shown in Fig. 10. The probability distribution of wind speed expressed by the Rayleigh distribution is quite different from the actual one; while the two parameter Weibull distribution model is obviously different from the three parameter distribution and the actual distribution in the zero and low wind speed regions, while the surface structure in the suburb of the city is complex, with the influence of buildings, trees and shrubs, and the rough length is approximately 167 times that of the grassland, 4000 times that of the sea. This obvious difference in retardation makes the probability of low velocity wind samples taking up the whole sample in the range of suburban near ground height quite considerable, it is necessary to require the corresponding probability distribution function to accurately represent the probability distribution of zero wind and low-speed wind interval. Compared with the above three types of probability distribution functions, only the three parameter Weibull distribution model has a good consistency with the actual wind speed distribution in the low wind speed interval. Moreover, from the results of the goodness test of four different standards (Tables 5 and 6), the three parameter Weibull distribution is more suitable for describing the probability distribution of wind speed within the test height than the other two distribution models.

**Table 4:** Values of *μ*, *k*, and *c* at each observation position.

Height range	10 m	11 m	12 m	13 m	14 m	15 m	16 m	17 m	18 m	19 m	38 m
*μ*	0.60	0.61	0.62	0.62	0.66	0.66	0.67	0.67	0.68	0.68	0.71
*k*	0.30	0.31	0.31	0.31	0.33	0.33	0.33	0.33	0.34	0.34	0.35
*c*	0.45	0.46	0.46	0.48	0.49	0.50	0.50	0.50	0.51	0.51	0.53

**Table 5:** Chi-square goodness and R^2^ of fit test.

Height	Simplify the Chi-square			R^2^		
	Rayleigh	Weibull2	Weibull3	Rayleigh	Weibull2	Weibull3
10 m	0.00104	8.82E−05	3.56E−05	0.98268	0.99853	0.99941
11 m	0.0012	3.18E−04	1.31E−05	0.98449	0.99592	0.99983
12 m	0.0012	3.18E−04	1.31E−05	0.98449	0.99592	0.99983
13 m	0.00103	3.89E−05	1.72E−05	0.97807	0.99917	0.99963
14 m	9.60E−04	3.40E−05	1.83E−05	0.97763	0.99921	0.99957
15 m	0.00128	4.09E−05	2.71E−05	0.97520	0.99921	0.99948
16 m	0.00102	3.70E−04	2.18E−05	0.98432	0.99626	0.99978
17 m	0.00107	6.10E−05	2.64E−05	0.97795	0.99875	0.99946
18 m	0.00126	6.20E−05	3.43E−05	0.97650	0.99885	0.99936
19 m	0.00118	0.00118	3.43E−05	0.97718	0.97718	0.99934
38 m	0.00104	8.82E−05	3.56E−05	0.98268	0.99853	0.99941

**Table 6:** RSS/SSE and RMSE test of goodness.

Height	RSS/SSE			RMSE		
	Rayleigh	Weibull2	Weibull3	Rayleigh	Weibull2	Weibull3
10 m	1.03593	0.08781	0.03553	0.004691997	0.001366043	0.000868941
11 m	1.20287	0.31624	0.01302	0.005121666	0.002626094	0.000532853
12 m	1.20287	0.31624	0.01302	0.005121666	0.002626094	0.000532853
13 m	1.02738	0.0387	0.01713	0.004705661	0.000913294	0.000607622
14 m	0.95879	0.03389	0.01824	0.00454083	0.000853708	0.000626305
15 m	1.28107	0.04077	0.027	0.005242264	0.000935196	0.000761052
16 m	0.97209	0.3681	0.0217	0.004560902	0.002806599	0.00068144
17 m	1.06774	0.06072	0.02627	0.004778441	0.001139513	0.00074952
18 m	1.26048	0.06171	0.03416	0.005191509	0.001148691	0.000854643
19 m	1.17745	1.17745	0.0342	0.005016162	0.005016162	0.000854896
38 m	1.03593	0.08781	0.03553	0.004691997	0.001366043	0.000868941

## Parameter analysis of wind speed distribution model

**Figure 11:**
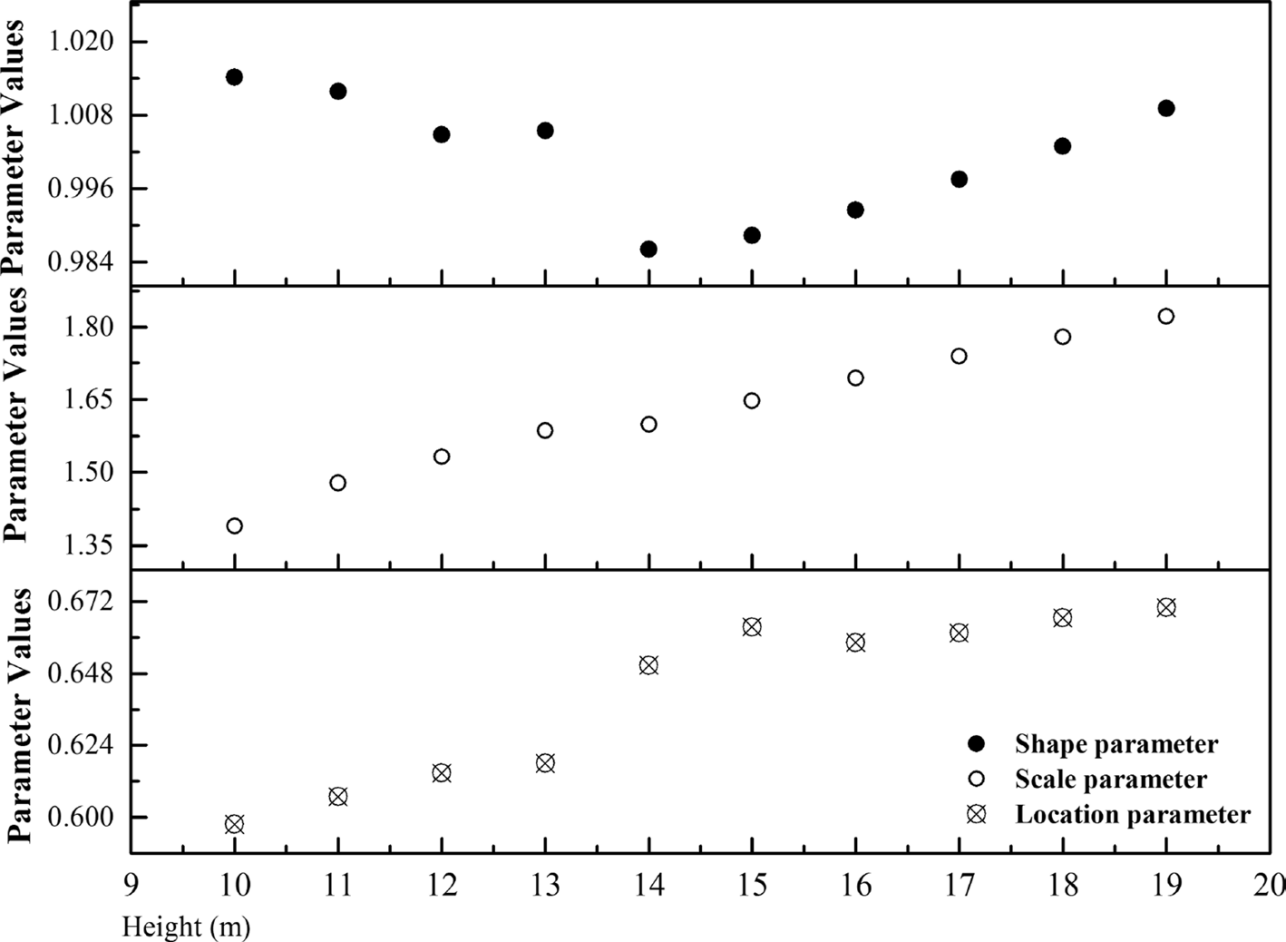
Parameter values of Weibull distribution function at each observed altitude.

The three unknown parameters of the three-parameter Weibull model are the shape parameter *k*, scale parameter *c*, and location parameter *μ*. Nonlinear regression analysis was used to estimate the values of these three parameters at 10 observation positions (Fig. 11). Its mathematical significance is that while *c* and *μ* are the same, *k* affects the shape change of the distribution curve. When *k* and *μ* are the same, *c* affects the size of the x-coordinate scale. When *k* and *c* are the same, *μ* determines the starting position of the curve.

The variation of location parameters and scale parameters with height presents a monotonically increasing trend (Fig. 11). However, the increasing range is not the same. Scale parameters increase by approximately 0.4 in the height range of 10–19 m. However, the increasing range of location parameters is only approximately 0.28. Therefore, in the scale range of 10–19 and 10 m, the influence of height growth on scale parameters is more significant than that of position parameters. However, no similar trend was observed in the variation of shape parameters. The variation was the smallest of the three parameters. The mathematical relevance of the corresponding parameters is that while height increases, the span between the wind speed values of the Weibull probability distribution function also increases because the three-parameter Weibull distribution function considers the influence of static wind. As the wind speed in the vertical direction of the gradient increases, the frequency of static winds decreases. This condition also indirectly proves that in the urban wind environment where static wind, low speed wind, and strong wind exist alternately, the three-parameter Weibull probability distribution model considering static wind factor has good fit with the local actual wind conditions of the city.

**Figure 12:**
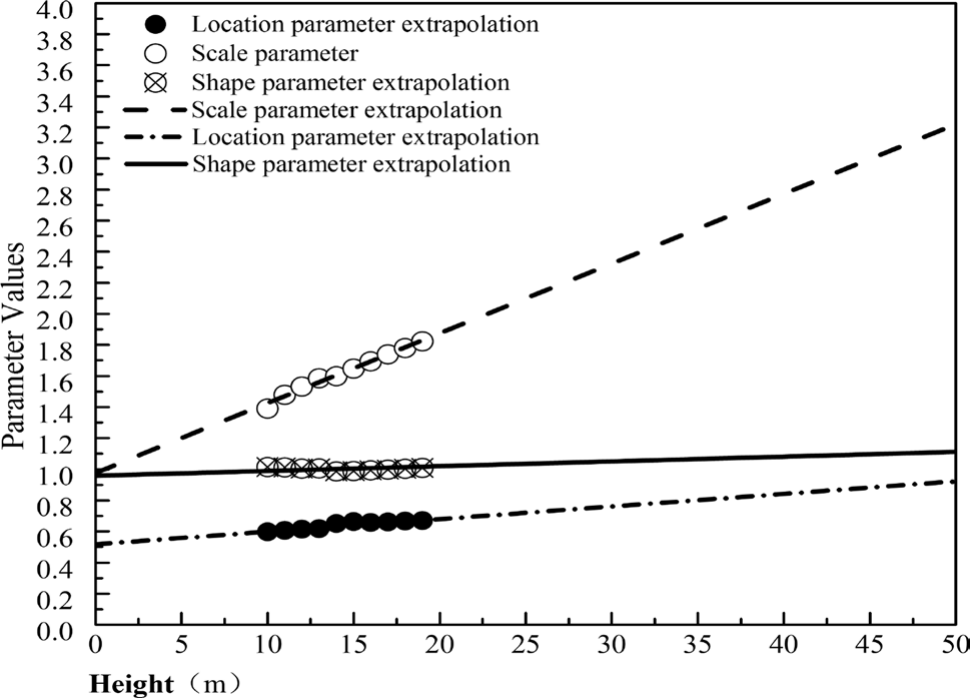
Value of the Weibull parameter (10–19 m) and the extrapolation value.

**Figure 13:**
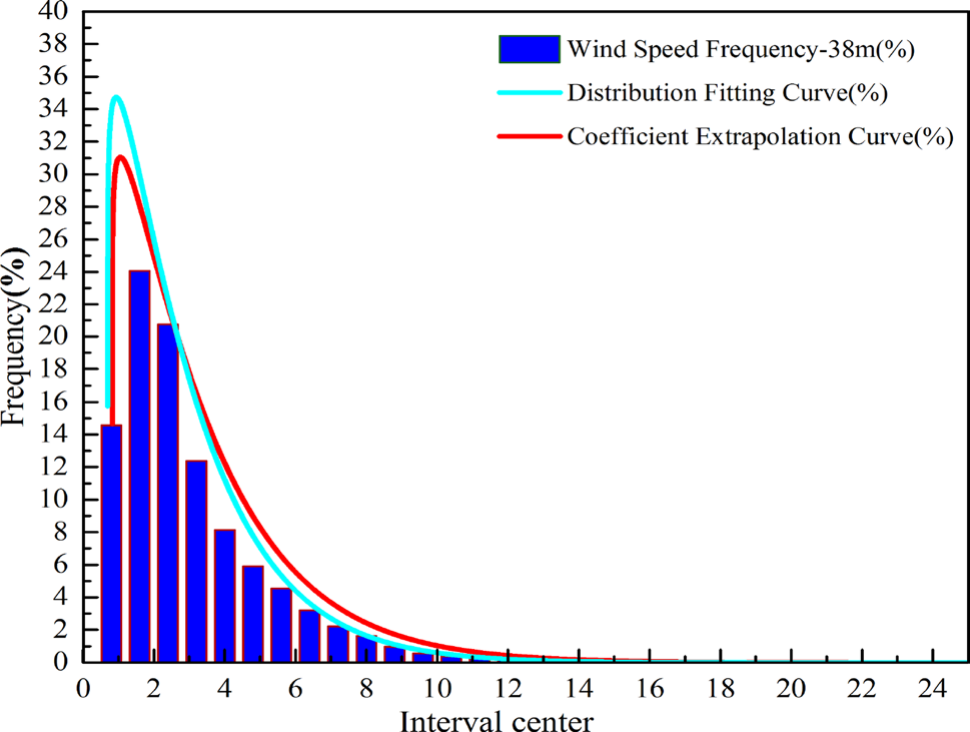
Probability distribution and extrapolation probability distribution curves of actual wind speed at 38 m.

**Figure 14:**
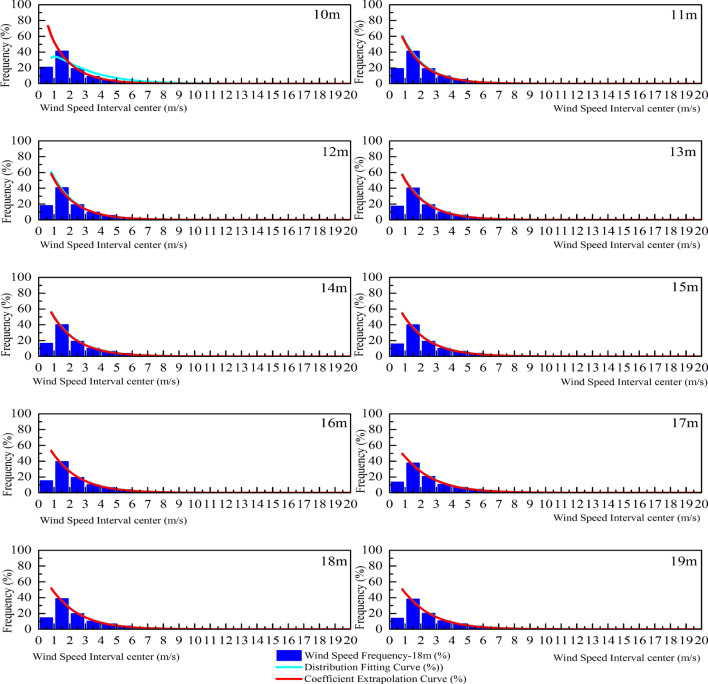
Probability distribution and extrapolation probability distribution curves of actual wind speed at 10–19 m.

Based on the Weibull distribution function model and corresponding parameter values obtained from the wind speed data at the 10 positions of 10–19 m, this study extrapolates the parameter values and wind speed distribution function model at other height positions (Fig. 12). The distribution function model and parameters of the relative height at 38 m are compared, and the results are shown in Fig. 13. See Fig. 14 for the comparison between the function constructed by extrapolation parameters at other heights and the actual distribution function.

The extrapolated model parameters are close to the actual wind speed probability distribution in the interval over 2 m/s. Thus, the extrapolated value at the height not reached by the probe can be used to qualitatively determine the overall probability distribution of wind speed at that point. However, the extrapolation between 0 and 2 m/s is lower than the actual frequency of wind speed, which may lead to an underestimation of wind potential.

Regardless of the scale, shape, or position parameters, the monthly changes at each observation height have maintained relatively consistent rules. However, the position and shape parameters have different vertical variation characteristics (10–19 m) in different months, and only the scale parameters show stable linear growth.

**Figure 15:**
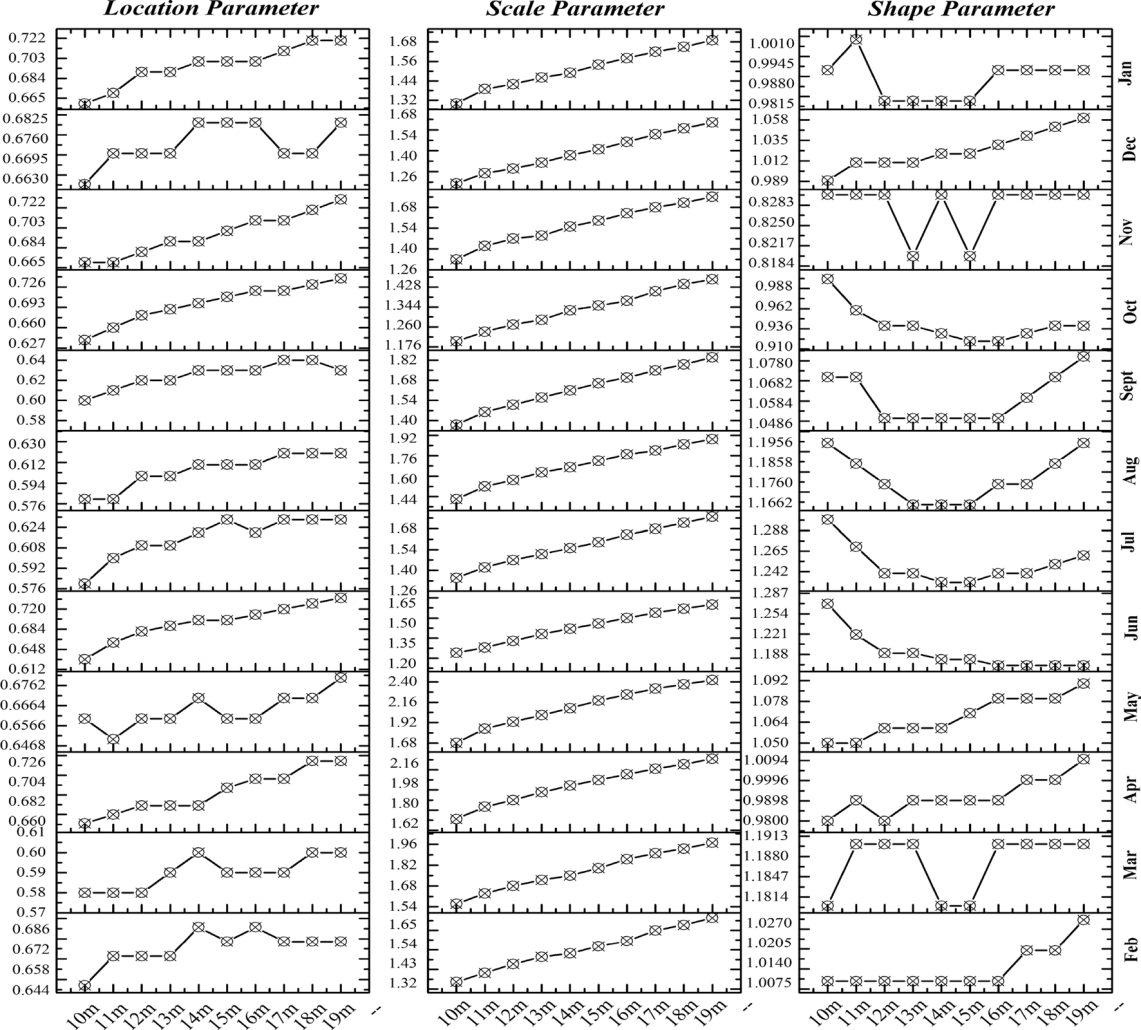
Parameter values at the observed position of 10–19 m (monthly).

**Figure 16:**
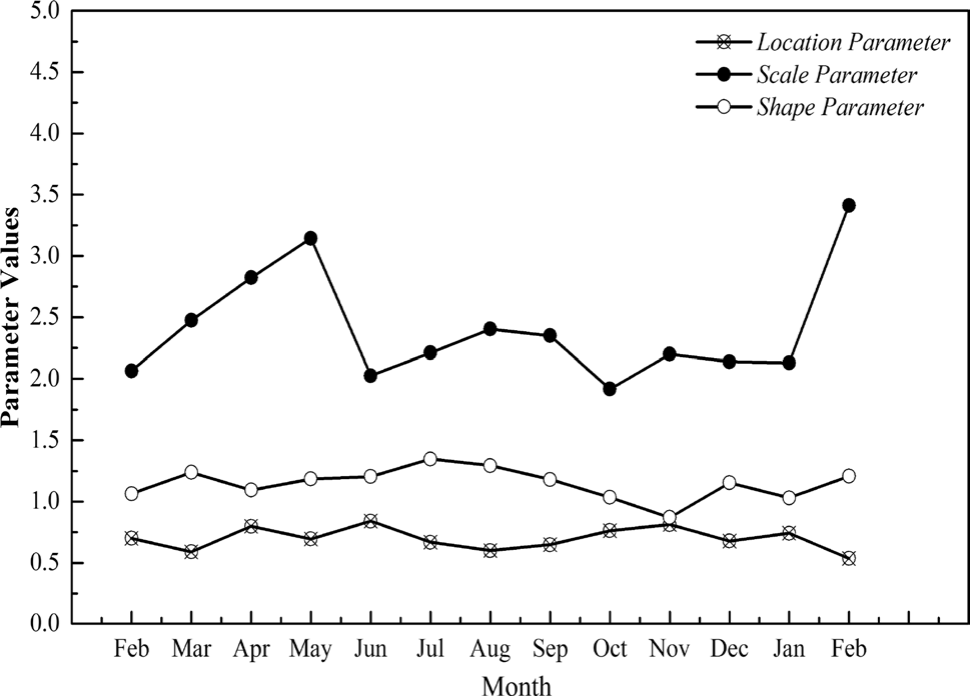
Parameter values at the observed position of 38 m (monthly).

To understand the relationship between seasons and the probability distribution of wind speed, this paper also summarizes and sorts the parameter values of the above three parameters in each month of the observation year. The parameter values have no significant seasonal characteristics and are not significantly correlated with meteorological parameters, such as air temperature, atmospheric pressure, and air density (Figs. 13, 14, and 15), and parameter values of the wind speed probability distribution model in the observed year, neither seasonally nor monthly. Moreover, the parameter values estimated using a year’s data affect the monthly variation (Fig. 15 and 16), making it easy to underestimate or overestimate the potential of wind energy in a dispersed and highly variable wind environment, such as urban area, where the observation site is located. Furthermore, in a built environment, such as energy base, the proportion of very low or zero wind speeds in the overall sample of monthly wind speeds does not change considerably. In addition, considering the mathematical significance of position parameter, scale parameter and shape parameter in Weibull distribution, when the wind speed data of each month is taken as the estimation, the difference of parameters of each month is mainly reflected in the scale parameter, which is directly related to the difference of wind speed threshold in different months. Figures 15 and 16 reflect the location parameters and shape parameters, from which we can see that the frequency of low wind speed interval and the frequency structure of the whole wind speed interval have little difference in each month of the year; The wind speed fluctuates greatly from February to may, and the wind speed threshold is the highest in the whole year. In other months, the wind speed keeps within a relatively stable range.

The scale parameter determines the threshold value of the wind speed interval. In other words, the larger the parameter, the larger the maximum wind speed value of the wind speed interval. Wind speed decreases in the vertical direction with the influence of the underlying surface, and the average wind speed in a certain time period generally increases in the form of power law or exponential law (Fig. 9). However, the scale parameter is related to the maximum wind speed in the wind speed data sample.

The shape parameter determines the shape of the probability density curve. According to the properties of Weibull distribution, when the shape parameter is 1, the Weibull distribution is simplified into an exponential distribution. When the annual wind speed data of 10 min is taken as a sample, the value of shape parameters at each height is close to 1, and the deviation is within ± 0.03. The distribution of wind speed tends to exponential distribution at this time. When taking the wind speed data of each month as the sample, although the whole is close to 1, the difference of some months is more than 0.3, which has changed the distribution form. Based on the same time span, statistical analysis of samples made throughout the year will affect the actual differences of each month. When conditions permit, separate statistics on monthly meteorological and wind speed data of the location of wind energy utilization in urban plans are conducive to the seasonal advantage of wind energy, which is also shown in the wind energy density estimation below.

## Assessment of wind resources

**Figure 17:**
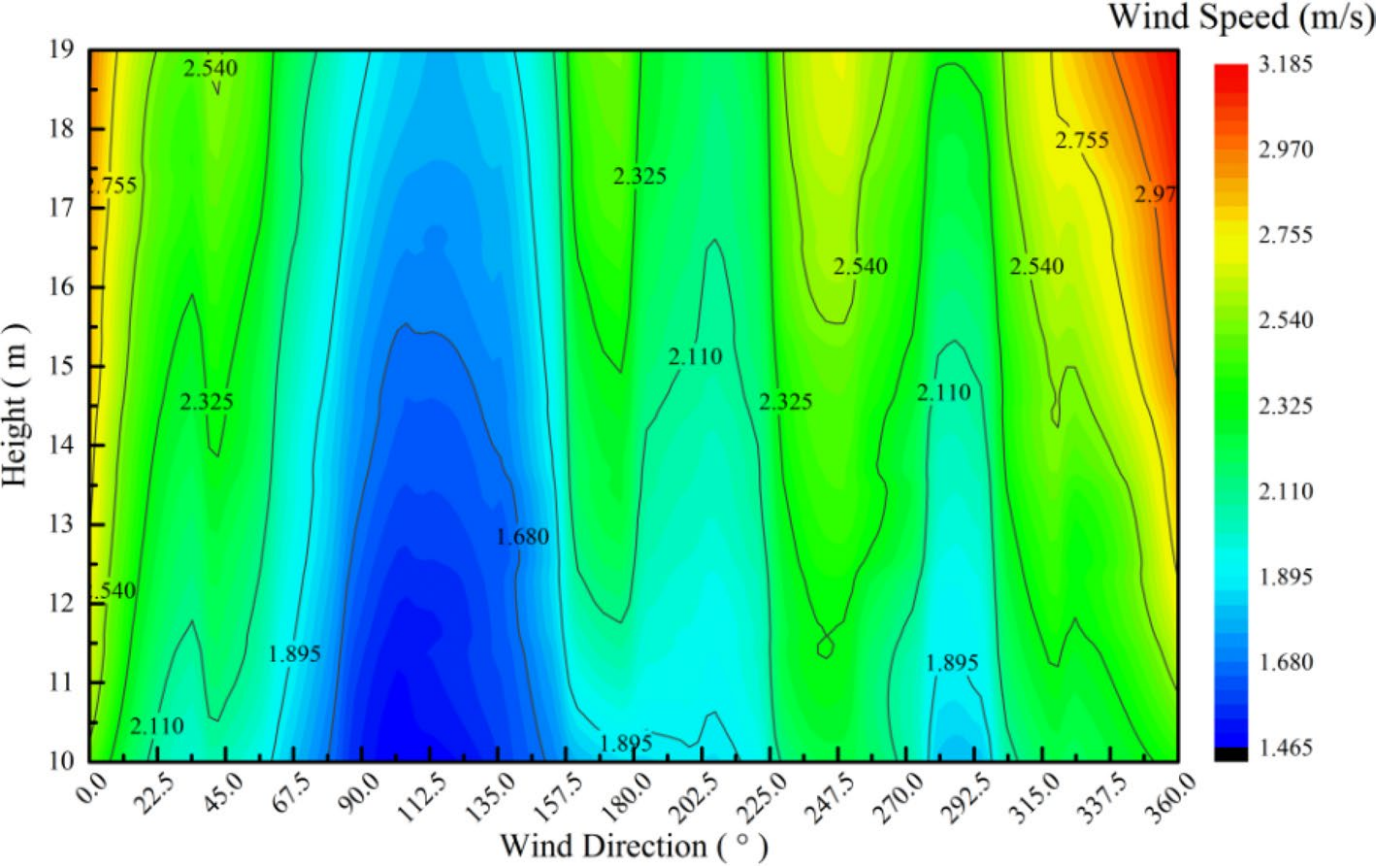
Annual wind speed distribution at all observed altitudes of 10–19 m.

The intensity distribution of the wind speed at all heights is almost the same in the 16 wind direction ranges. The speed in the direction of NNE is lower, whereas the wind speed in the direction of N is higher (Fig. 17).

According to the change of wind direction, the steady distribution of wind direction between vertical layers is suitable for installing vertical axis wind turbine. For the horizontal axis wind turbine, attention should be paid to the effect of yaw on the operation of the wind turbine. However, an evident gradient is observed in the wind speed from the 16 directions (Fig. 17), which will add a large non-uniform wind load to the wind wheel and central axis and affect the safe and stable operation of the wind turbine.

**Figure 18:**
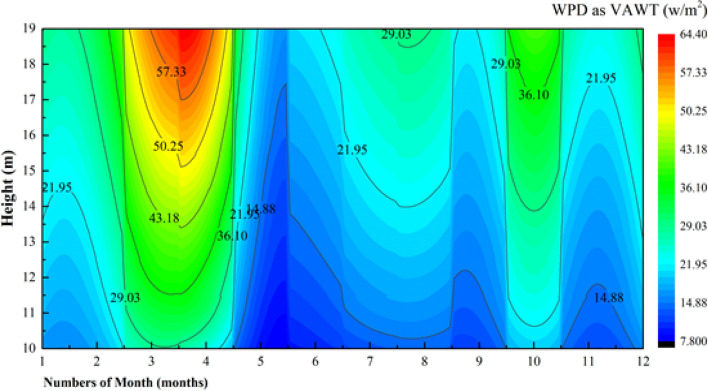
Wind energy density (VAWT) per month at 10–19 m.

**Figure 19:**
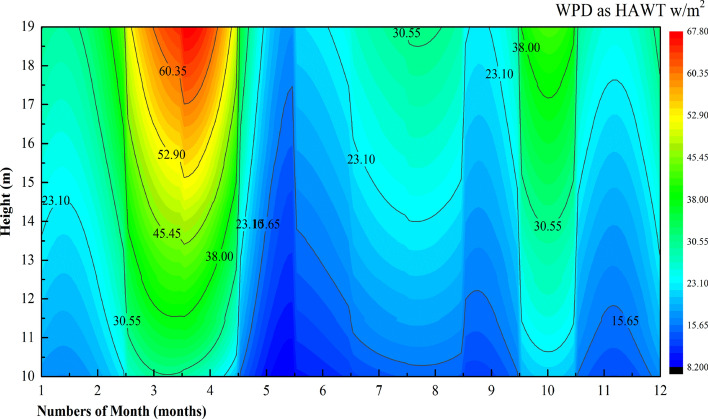
Wind energy density (HAWT) per month at 10–19 m.

**Figure 20:**
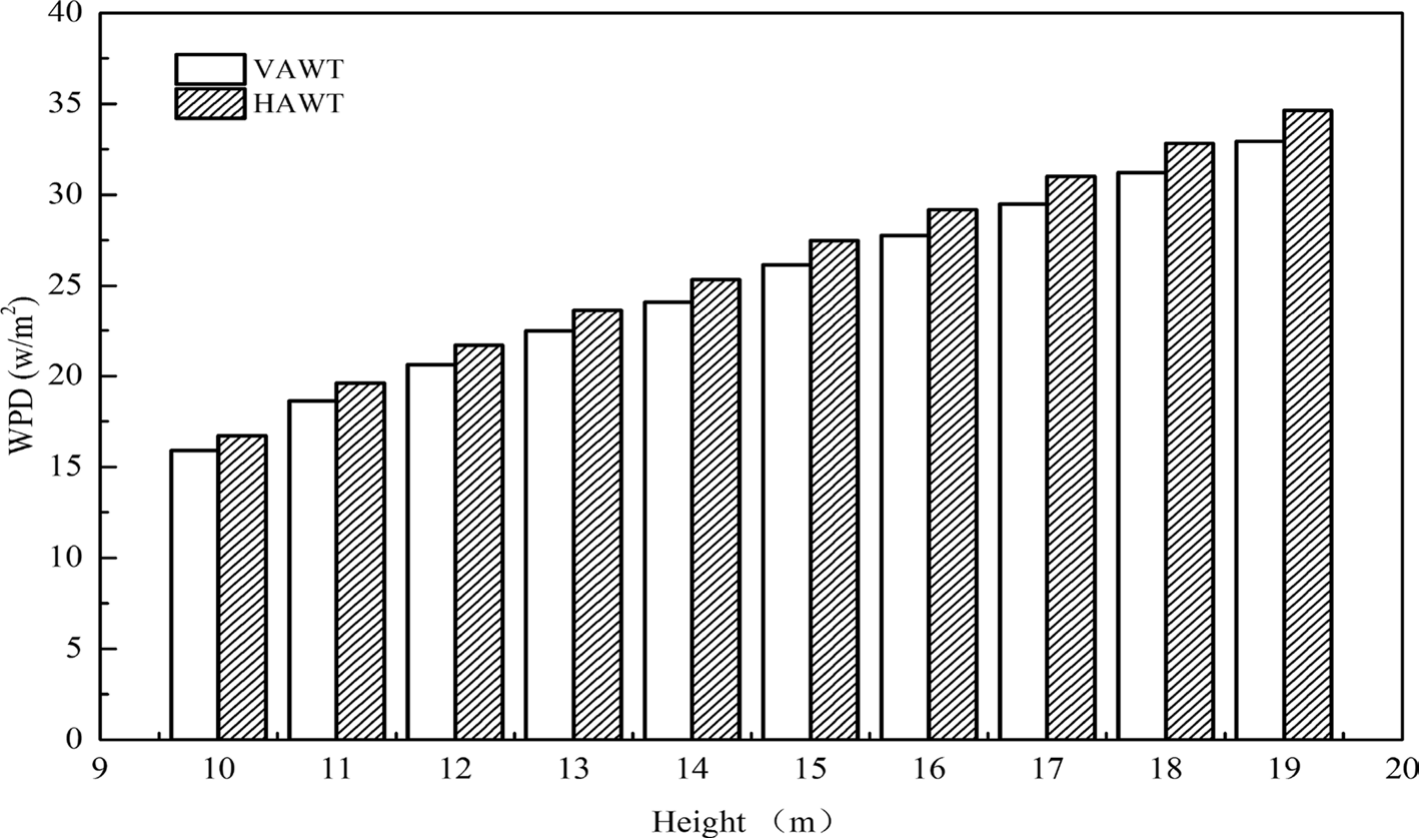
Annual wind density at each height.

To further evaluate the wind energy potential at the observation position, the basic parameters of two small vertical axis and horizontal axis wind turbines (Table 7) were selected to calculate the wind energy density. Spring is the season with the highest wind density, followed by autumn (Figs. 18 and 19). At 10–19 m, the horizontal axis (Fig. 20) is better than the vertical axis but not enough to compensate for the effect of yaw.

**Table 7:** Basic parameters of commercial horizontal and vertical axis wind turbines.

Wind turbine	Rated power (W)	Rotor diameter (m)	Blade length/height (m)	Start-up wind speed (m/s)
H-300W (vertical axis)	300	1.2	1.4	2
FD1.5-300W (horizontal axis)	300	1.5	0.7	2.5

In *IEC61400-1*, if a turbine is installed, it will greatly affect the stability and safety of the wind turbine. The power law change of TI measured by Lidar in the vertical direction conforms to the curve rule of the function shown in Eq. (19).

19 $$l\left(h\right)=0.2{e}^{\left(\frac{6.44}{x+3.66}\right)}$$

**Figure 21:**
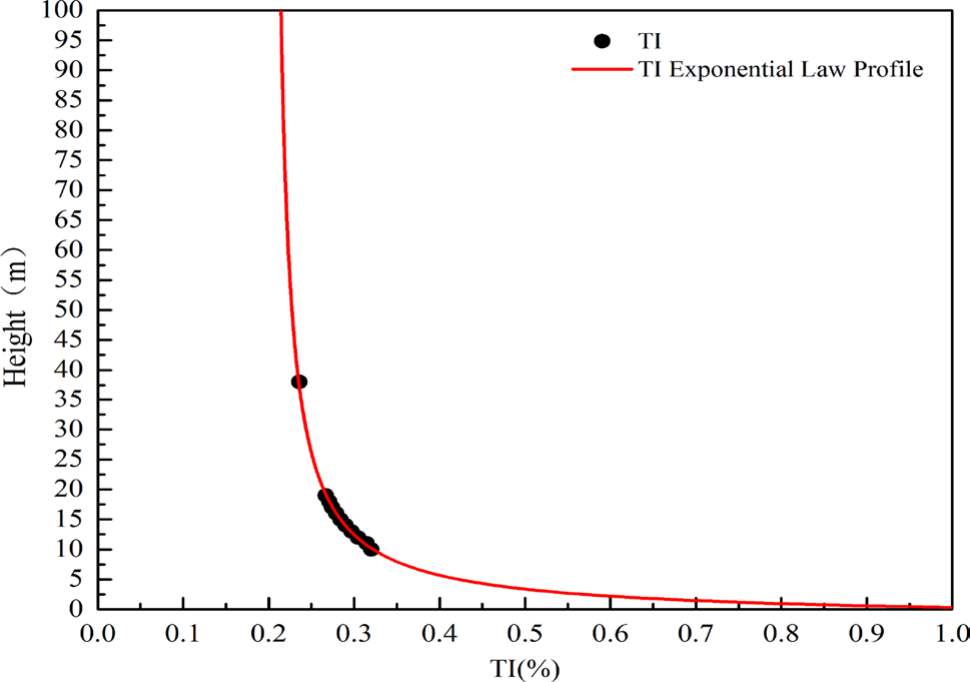
TI vertical variation law.

**Figure 22:**
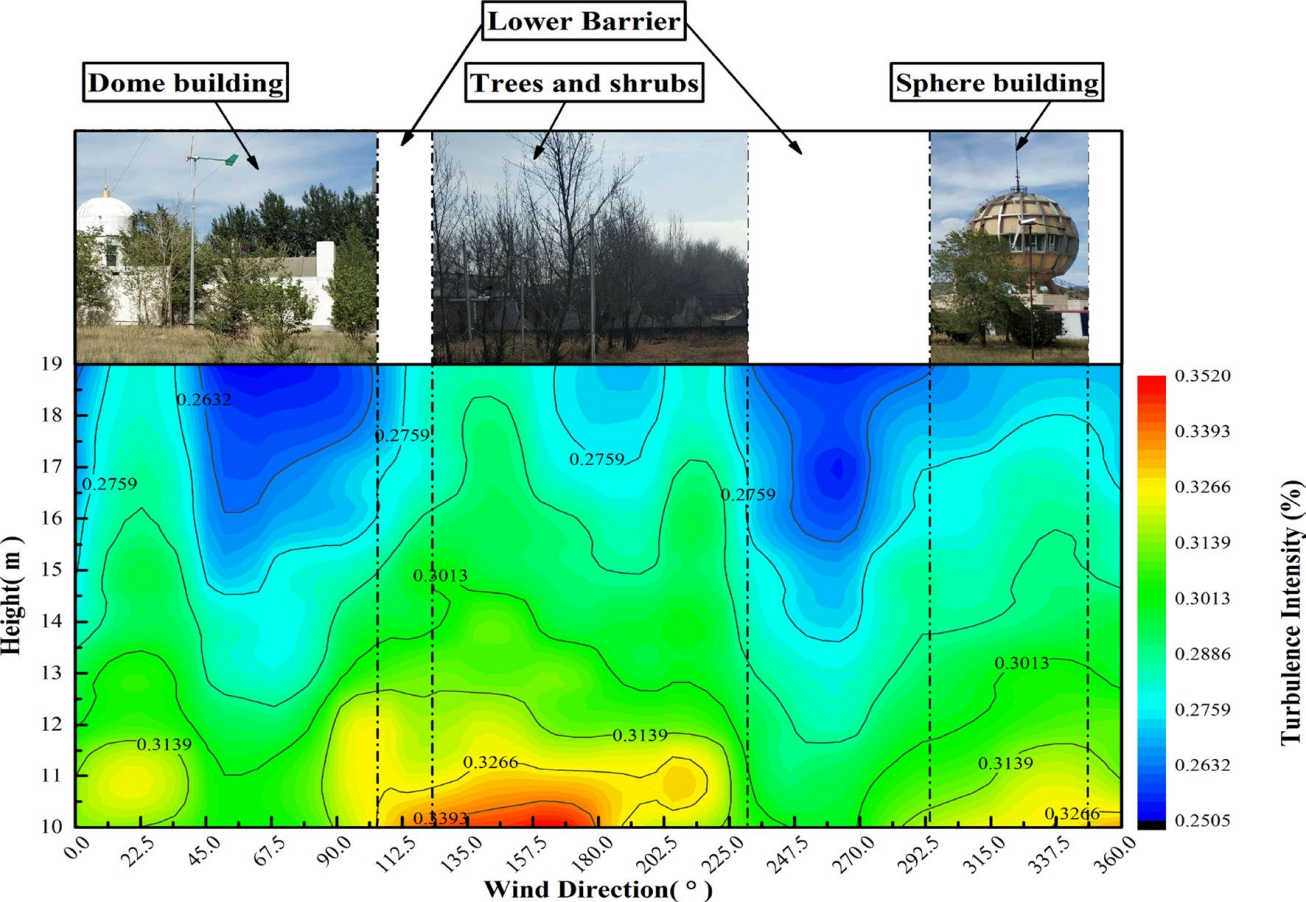
10–19 m TI is distributed by wind direction throughout the year at all observed altitudes.

Although we are not quite sure about the size of extrapolate TI beyond 38, but the simulated extrapolation curve of TI at the actual height also indicates that it is difficult for TI to be less than 20% at the height of 38–10 m (Fig. 21). However, after classifying the TI at these observed heights according to 16 wind direction intervals (Fig. 22), the TI has a relatively regular expression in the wind direction. In the area where TI is relatively high [90, 225], the incoming flow direction is a continuous row of shrubs with a height of approximately 20 m in the south of the observation site. By contrast, buildings in the northeast and northwest of the site have no such effect on turbulence near the site.

## Conclusions

The continuous promotion of urban wind energy utilization requires not only theoretical research and innovation of energy utilization model, but also diverse field data to understand the wind environment characteristics of microclimate groups in different urban scales and different urban areas.

In this study, ZephIR Lidar was used to observe the wind environment indicators 10–19 m above the relatively open area of an experimental base in the urban outskirts of Hohhot for one year. The Weibull distribution function was used to analyze its statistical characteristics. The results showed that the three-parameter Weibull probability distribution model has good applicability to the wind speed characteristics of urban suburban buildings similar to the energy bases. Therefore, the spatial and temporal variation of the parameters and the estimation of wind energy density were discussed, and the following conclusions were drawn:

1. In this paper, Eqs. (18) and (19), the exponential form based on natural logarithm, can well fit the vertical changes of wind speed and TI in the station. Within the observed height range, 12–19 m is a relatively stable wind-shear height zone.
2. From the perspective of the sample source of wind data and the characteristics of its Weibull distribution, capturing the dispersed and changing wind energy in cities by fitting parameters of each month alone is beneficial. The scale, shape, and position parameters generally change linearly within the height of 10–19 m. The wind speed probability distribution at 38 m, which is composed of the parameter values derived in extrapolation, at the range of 0–2 m/s is significantly lower than the probability value of the actual distribution at this position. Thus, the wind speed region within 0–2 m/s is underestimated. However, the limitations of urban observation conditions can still be solved on the whole.
3. In terms of the available wind potential, the annual wind density of the 300 W commercial horizontal axis wind turbine in the range of 10–19 m is 61.73 W/m^2^, whereas the vertical axis wind density is 58.71 W/m^2^. However, the wind direction statistics at the observation site show that the environment is suitable for installing vertical axis wind turbine (not including the roof). Wind energy is abundant in April and May, 60.68 and 63.38 W/m^2^, respectively. These values are approximately three times of the month with the lowest wind energy density. Moreover, in the environment where the site is located, approximately 100 W wind turbines seem to be suitable.
4. The TI near the station is still hardly lower than 20% within 0–100 m. However, the TI in the wind direction with trees in the incoming flow direction is higher by approximately 1% than that in the direction of buildings.

## Data Availability

The authors would like to acknowledge the usage of the ZephIR Lidar provided by the laboratory of utilization mechanism and optimization of wind energy and solar energy of college of energy and power Engineering of the Inner Mongolia University of Technology.
